# Supplementary material for: Impact of COVID-19 Pandemic on Management and Outcomes in Patients with Septic Shock in the Emergency Department
Source: J Pers Med. 2022 Nov 1;12(11):1803. doi: 10.3390/jpm12111803 (PMC9695029; doi:10.3390/jpm12111803)
Supplement: Supplementary file 1 [file jpm-12-01803-s001.zip › table_s1.pdf]

# Supplementary Material

**Table S1.** Hospital based clinical outcomes – unmatched cohort

| | Hospital A | | | | Hospital B | | | |
|---|---|---|---|---|---|---|---|---|
| | Overall (N=9) | Pre-COVID-19 (N=9) | Post-COVID-19 (N=0) | *p-va*lue | Overall (N=119) | Pre-COVID-19 (N=95) | Post-COVID-19 (N=24) | *p-va*lue |
| In-hospital mortality, NO. (%) | 6 (66.70) | 6 (66.70) | - | - | 25 (21.00) | 19 (20.00) | 6 (25.00) | 0.80 |
| 28-day mortality, NO. (%) | 6 (66.70) | 6 (66.70) | - | - | 23 (19.30) | 19 (20.0) | 4 (16.70) | 0.39 |
| ED length of stay (h), median [IQR] | 2.68 [2.60–5.13] | 2.68 [2.60–5.13] | - | - | 12.22 [7.47–27.08] | 11.52 [7.23–27.39] | 13.83 [8.82–24.60] | 0.60 |
| ICU-admitted patients' ED length of stay (h), median [IQR] | 2.68 [2.60–5.13] | 2.68 [2.60–5.13] | - | - | 9.95 [6.77–22.04] | 9.95 [6.77–22.04] | 9.53 [8.01–15.36] | 0.95 |
| | Hospital C | | | | Hospital D | | | |
| | Overall (N=347) | Pre-COVID-19 (N=229) | Post-COVID-19 (N=118) | *p*-value | Overall (N=142) | Pre-COVID-19 (N=73) | Post-COVID-19 (N=69) | *p*-value |
| In-hospital mortality, NO. (%) | 104 (30.00) | 61 (26.60) | 43 (36.40) | 0.08 | 35 (24.60) | 15 (20.50) | 20 (29.00) | 0.33 |
| 28-day mortality, NO. (%) | 98 (28.20) | 58 (25.30) | 40 (33.90) | 0.10 | 33 (23.20) | 15 (20.50) | 18 (26.10) | 0.32 |
| ED length of stay (h), median [IQR] | 6.53 [4.78–9.62] | 6.38 [4.77–8.77] | 7.34 [4.80–14.52] | 0.09 | 7.78 [5.62–12.78] | 6.45 [5.37–11.62] | 9.18 [6.62–14.13] | 0.01 |
| ICU-admitted patients' ED length of stay (h), median [IQR] | 6.53 [4.87–9.53] | 6.40 [4.86–8.44] | 7.20 [4.88–15.43] | 0.11 | 7.32 [5.59–11.05] | 5.88 [5.19–8.50] | 9.05 [6.62–13.08] | <0.001 |
| | Hospital E | | | | Hospital F | | | |
| | Overall (N=74) | Pre-COVID-19 (N=66) | Post-COVID-19 (N=8) | *p*-value | Overall (N=75) | Pre-COVID-19 (N=51) | Post-COVID-19 (N=24) | *p*-value |
| In-hospital mortality, NO. (%) | 21 (28.40) | 18 (27.30) | 3 (37.50 | 0.85 | 17 (22.70) | 11 (21.60) | 6 (25.00) | 0.97 |
| 28-day mortality, NO. (%) | 24 (32.40) | 20 (30.30) | 4 (50.00) | 0.18 | 16 (21.30) | 9 (17.60) | 7 (29.20) | 0.01 |
| ED length of stay (h), median [IQR] | 10.78 [7.00–21.22] | 11.79 [7.03–21.22] | 8.87 [6.22–21.58] | 0.70 | 7.43 [5.30–16.96] | 7.20 [5.30–16.61] | 7.67 [5.61–16.12] | 0.90 |
| ICU-admitted patients' ED length of stay (h), median [IQR] | 8.63 [6.80–10.84] | 8.31 [6.80–10.84] | 8.87 [7.83–11.79] | 0.81 | 6.10 [4.53–8.12] | 5.80 [4.52–7.58] | 6.77 [5.05–10.27] | 0.43 |
| | Hospital G | | | | Hospital H | | | |
| | Overall (N=1337) | Pre-COVID-19 (N=891) | Post-COVID-19 (N=446) | *p*-value | Overall (N=176) | Pre-COVID-19 (N=109) | Post-COVID-19 (N=67) | *p*-value |
| In-hospital mortality, NO. (%) | 313 (23.40) | 211 (23.70) | 102 (22.90) | 0.80 | 42 (23.90) | 26 (23.90) | 16 (23.90) | 1.00 |
| 28-day mortality, NO. (%) | 317 (23.70) | 215 (24.10) | 102 (22.90) | 0.78 | 41 (23.30) | 29 (26.60) | 12 (17.90) | 0.25 |
| ED length of stay (h), median [IQR] | 12.35 [6.82–22.47] | 11.13 [6.16–22.80] | 14.37 [8.70–22.35] | <0.001 | 7.35 [4.41–11.42] | 6.15 [4.18–9.12] | 9.80 [5.93–13.69] | 0.001 |

| | | | | | | | | |
|---|---|---|---|---|---|---|---|---|
| ICU-admitted patients' ED length of stay (h), median [IQR] | 7.23 [4.99–11.08] | 6.85 [4.80–10.40] | 8.63 [5.72–13.02] | <0.001 | 7.36 [4.39–11.49] | 6.15 [4.11–9.02] | 9.80 [5.95–13.98] | 0.001 |
| | Hospital I | | | | Hospital J | | | |
| | Overall (N=688) | Pre-COVID-19 (N=530) | Post-COVID-19 (N=158) | *p*-value | Overall (N=219) | Pre-COVID-19 (N=172) | Post-COVID-19 (N=47) | *p*-value |
| In-hospital mortality, NO. (%) | 145 (21.10) | 113 (21.30) | 32 (20.30) | 0.86 | 47 (21.50) | 36 (20.90) | 11 (23.40) | 0.87 |
| 28-day mortality, NO. (%) | 133 (19.30) | 104 (19.60) | 29 (18.40) | 0.81 | 38 (17.40) | 27 (15.70) | 11 (23.40) | 0.05 |
| ED length of stay (h), median [IQR] | 10.15 [6.70–17.94] | 9.50 [6.17–16.82] | 12.57 [8.09–19.55] | <0.001 | 16.00 [9.72–28.68] | 17.20 [10.25–30.24] | 12.67 [8.77–19.77] | 0.01 |
| ICU-admitted patients' ED length of stay (h), median [IQR] | 7.82 [5.40–11.70] | 7.59 [5.29–11.25] | 9.65 [7.03–12.92] | 0.003 | 15.23 [9.97–23.50] | 15.48 [10.14–24.49] | 10.86 [8.28–15.32] | 0.19 |
| | Hospital K | | | | Hospital L | | | |
| | Overall (N=466) | Pre-COVID-19 (N=321) | Post-COVID-19 (N=145) | *p*-value | Overall (N=8) | Pre-COVID-19 (N=8) | Post-COVID-19 (N=0) | *p*-value |
| In-hospital mortality, NO. (%) | 123 (26.40) | 77 (24.00) | 46 (31.70) | 0.10 | 1 (12.50) | 1 (12.50) | - | - |
| 28-day mortality, NO. (%) | 113 (24.20) | 75 (23.40) | 38 (26.20) | 0.17 | 1 (12.50) | 1 (12.50) | - | - |
| ED length of stay (h), median [IQR] | 8.17 [5.62–13.42] | 7.50 [5.45–12.28] | 9.42 [6.00–15.92] | 0.003 | 5.01 [3.95–6.38] | 5.01 [3.95–6.38] | - | - |
| ICU-admitted patients' ED length of stay (h), median [IQR] | 7.45 [5.32–11.09] | 7.02 [5.20–9.50] | 8.99 [5.83–13.55] | 0.001 | 5.01 [3.95–6.38] | 5.01 [3.95–6.38] | - | - |
| | Hospital M | | | | Hospital N | | | |
| | Overall (N=8) | Pre-COVID-19 (N=0) | Post-COVID-19 (N=8) | *p*-value | Overall (N=29) | Pre-COVID-19 (N=0) | Post-COVID-19 (N=29) | *p*-value |
| In-hospital mortality, NO. (%) | 5 (62.50) | - | 5 (62.50) | - | 4 (13.80) | - | 4 (13.80) | - |
| 28-day mortality, NO. (%) | 4 (50.00) | - | 4 (50.00) | - | 2 (6.90) | - | 2 (6.90) | - |
| ED length of stay (h), median [IQR] | 11.21 [9.13–16.69] | - | 11.21 [9.13–16.69] | - | 10.47 [7.23–20.92] | - | 10.47 [7.23–20.92] | - |
| ICU-admitted patients' ED length of stay (h), median [IQR] | 9.68 [7.48–10.45] | - | 9.68 [7.48–10.45] | - | 10.33 [7.47–27.83] | - | 10.33 [7.47–27.83] | - |

COVID-19, coronavirus disease 2019; ED, emergency department; ICU, intensive care unit.
