# Supplementary material for: Impact of COVID-19 Pandemic on Management and Outcomes in Patients with Septic Shock in the Emergency Department
Source: J Pers Med. 2022 Nov 1;12(11):1803. doi: 10.3390/jpm12111803 (PMC9695029; doi:10.3390/jpm12111803)
Supplement: Supplementary file 1 [file jpm-12-01803-s001.zip › table_s2.pdf]

Supplementary Material

Table S2. Hospital based septic shock related interventions – unmatched cohort

Septic shock related interventions	Hospital A				Hospital B			
	Overall (N=9)	Pre-COVID-19 (N=9)	Post-COVID-19 (N=0)	*p-va*lue	Overall (N=119)	Pre-COVID-19 (N=95)	Post-COVID-19 (N=24)	*p-va*lue
Time to lactate measurement (min), median [IQR]	5.00 [1.00–14.00]	5.00 [1.00–14.00]	-	-	30.00 [16.50–67.50]	27.00 [16.50–61.50]	38.00 [16.75–75.75]	0.44
Time to blood culture (min), median [IQR]	996.00 [893.00–1058.00]	996.00 [893.00–1058.00]	-	-	13.00 [6.00–45.00]	16.00 [7.00–73.50]	11.00 [0.00–14.25]	0.002
Blood culture within 1 h, NO. (%)	9 (100.00)	9 (100.00)	-	-	91 (76.50)	67 (70.50)	24 (100.00)	0.01
Blood culture within 3 h, NO. (%)	9 (100.00)	9 (100.00)	-	-	106 (89.10)	82 (86.30)	24 (100.00)	0.12
Time to IV antibiotics (min), median [IQR]	117.00 [84.00–160.00]	117.00 [84.00–160.00]	-	-	181.00 [135.00–243.00]	194.00 [152.00–257.50]	137.00 [113.00–158.50]	<0.001
IV antibiotics within 1 h, NO. (%)	2 (22.20)	2 (22.20)	-	-	5 (4.20)	2 (2.10)	3 (12.50)	0.09
IV antibiotics within 3 h, NO. (%)	7 (77.80)	7 (77.80)	-	-	58 (48.70)	37 (38.90)	21 (87.50)	<0.001
Fluid resuscitation within 3 h, NO. (%)	8 (88.90)	8 (88.90)	-	-	109 (91.60)	87 (91.60)	22 (91.70)	0.65
Time to vasopressor (min)*, median [IQR]	24.00 [12.00–97.00]	24.00 [12.00–97.00]	-	-	136.50 [92.50–210.25]	143.00 [92.00–227.00]	105.00 [95.50–147.50]	0.17
Vasopressor within 1 h*, NO. (%)	5 (55.60)	5 (55.60)	-	-	16 (13.60)	15 (15.80)	1 (4.30)	0.27
Vasopressor within 3 h*, NO. (%)	8 (88.90)	8 (88.90)	-	-	75 (63.60)	55 (57.90)	20 (87.00)	0.02
Time to source control (h)**, median [IQR]	-	-	-	-	10.33 [1.67–22.96]	10.33 [3.73–25.51]	11.05 [7.68–15.28]	0.68
Septic shock related interventions	Hospital C				Hospital D			
	Overall (N=347)	Pre-COVID-19 (N=229)	Post-COVID-19 (N=118)	*p-va*lue	Overall (N=142)	Pre-COVID-19 (N=73)	Post-COVID-19 (N=69)	*p-va*lue
Time to lactate measurement (min), median [IQR]	21.00 [11.00–45.00]	19.00 [11.00–42.00]	25.00 [14.00–50.75]	0.04	63.50 [44.25–84.50]	59.00 [47.00–75.00]	71.00 [37.00–100.00]	0.35
Time to blood culture (min), median [IQR]	197.00 [119.00–305.75]	193.50 [115.25–307.50]	199.50 [130.50–300.50]	0.50	203.50 [120.50–307.25]	212.00 [110.00–318.00]	201.00 [126.00–297.00]	0.76

Blood culture within 1 h, NO. (%)	27 (8.30)	23(10.60)	4(3.70)	0.06	9.00 (6.30)	8.00 (11.00)	1 (1.40)	0.05
Blood culture within 3 h, NO. (%)	150 (46.00)	106(48.60)	44(40.70)	0.22	62.00(43.70)	31.00 (42.50)	31 (44.90)	0.90
Time to IV antibiotics (min), median [IQR]	131.50 [87.50–213.00]	140.00 [92.50–225.00]	123.00 [79.00–185.00]	0.07	138.50 [75.00–228.50]	137.00 [73.00–205.00]	139.00 [92.00–295.00]	0.30
IV antibiotics within 1 h, NO. (%)	40 (11.80)	26(11.70)	14(12.00)	1.00	19.00 (13.40)	9.00 (12.30)	10 (14.50)	0.90
IV antibiotics within 3 h, NO. (%)	228 (67.10)	142(63.70)	86(73.50)	0.09	91.00 (64.10)	49.00 (67.10)	42 (60.90)	0.55
Fluid resuscitation within 3 h, NO. (%)	269 (77.50)	171(74.70)	98(83.10)	0.03	91.00 (64.10)	52.00 (71.20)	39 (56.50)	0.02
Time to vasopressor (min)*, median [IQR]	116.50 [61.75–221.50]	126.00 [69.50–242.00]	96.00 [54.00–190.00]	0.07	148.00 [97.00–220.00]	147.00 [100.00–218.50]	154.00 [91.50–228.25]	0.91
Vasopressor within 1 h*, NO. (%)	66 (23.90)	38(21.20)	28(28.90)	0.20	14.00 (10.90)	3.00 (4.80)	11 (16.70)	0.06
Vasopressor within 3 h*, NO. (%)	186 (67.40)	118(65.90)	68(70.10)	0.57	78.00 (60.50)	41.00 (65.10)	37 (56.10)	0.39
Time to source control (h)**, median [IQR]	27.13 [17.91–81.92]	26.42 [13.17–53.28]	69.88 [23.90–175.94]	0.01	62.63 [18.75–179.67]	49.28 [18.73–228.93]	129.55 [59.58–160.07]	0.63

Septic shock related interventions	Hospital E				Hospital F			
	Overall (N=74)	Pre-COVID-19 (N=66)	Post-COVID-19 (N=8)	*p-va*lue	Overall (N=75)	Pre-COVID-19 (N=51)	Post-COVID-19 (N=24)	*p-va*lue
Time to lactate measurement (min), median [IQR]	36.00 [24.00–54.50]	39.00 [27.25–55.00]	22.00 [16.50–25.25]	0.02	32.00 [23.00–69.00]	30.00 [23.00–60.50]	35.50 [20.75–83.50]	0.65
Time to blood culture (min), median [IQR]	223.00 [141.00–384.00]	228.00 [141.50–380.50]	202.00 [133.50–398.50]	0.98	105.00 [58.00–158.00]	107.00 [48.00–159.00]	103.50 [60.50–130.75]	0.93
Blood culture within 1 h, NO. (%)	10.00 (14.50)	9 (14.50)	1 (14.30)	1	22 (30.10)	16 (32.70)	6 (25.00)	0.69
Blood culture within 3 h, NO. (%)	28 (40.60)	25(40.30)	3 (42.90)	1	59 (80.80)	39 (79.60)	20 (83.30)	0.95
Time to IV antibiotics (min), median [IQR]	93.00 [29.00–185.00]	101.00 [32.50–236.00]	72.50 [13.75–102.50]	0.156	156.00 [100.00–224.25]	155.50 [93.00–219.50]	170.00 [112.50–225.00]	0.50
IV antibiotics within 1 h, NO. (%)	27 (38.00)	23 (36.50)	4 (50.00)	0.723	2 (2.70)	1 (2.00)	1 (4.20)	1.00
IV antibiotics within 3 h, NO. (%)	53 (74.60)	45 (71.40)	8 (100.00)	0.187	43 (58.10)	31 (62.00)	12 (50.00)	0.47
Fluid resuscitation within 3 h, NO. (%)	47 (63.50)	42 (63.60)	5 (62.50)	0.869	58 (77.30)	40 (78.40)	18 (75.00)	0.14
Time to vasopressor (min)*, median [IQR]	154.00 [89.00–284.00]	154.00 [84.00–267.50]	175.50 [127.75–326.00]	0.583	105.00 [49.50–176.50]	101.00 [57.00–162.00]	113.50 [35.75–184.75]	0.95

Vasopressor within 1 h, NO. (%)	8 (16.30)	7 (16.30)	1 (16.70)	1	20 (28.20)	12 (25.50)	8 (33.30)	0.68
Vasopressor within 3 h, NO. (%)	28 (57.10)	25 (58.10)	3 (50.00)	1	54 (76.10)	36 (76.60)	18 (75.00)	1.00
Time to source control (h), median [IQR]	13.75 [6.36–30.73]	13.75 [6.36–30.73]	-	-	7.99 [6.32–13.44]	7.47 [6.15–16.43]	9.37 [9.37–9.37]	0.66
Septic shock related interventions	Hospital G				Hospital H			
	Overall (N=1337)	Pre-COVID-19 (N=891)	Post-COVID-19 (N=446)	*p*-value	Overall (N=176)	Pre-COVID-19 (N=109)	Post-COVID-19 (N=67)	*p*-value
Time to lactate measurement (min), median [IQR]	37.00 [15.00–82.00]	32.00 [13.00–73.00]	48.50 [22.00–93.75]	<0.001	21.00 [3.75–61.00]	21.00 [7.00–91.00]	19.00 [0.00–48.50]	0.07
Time to blood culture (min), median [IQR]	103.00 [61.00–173.00]	96.00 [58.00–161.50]	125.50 [72.25–186.75]	<0.001	45.50 [23.00–258.25]	43.00 [21.00–173.00]	51.00 [28.00–486.50]	0.06
Blood culture within 1 h, NO. (%)	332 (24.80)	252 (28.30)	80 (17.90)	<0.001	97 (55.70)	62 (57.90)	35 (52.20)	0.56
Blood culture within 3 h, NO. (%)	1032 (77.20)	705 (79.10)	327 (73.30)	0.02	119 (68.40)	80 (74.80)	39 (58.20)	0.03
Time to IV antibiotics (min), median [IQR]	135.00 [82.00–207.00]	127.00 [79.25–204.00]	150.00 [92.00–213.75]	0.002	138.50 [67.25–257.00]	142.00 [64.00–249.00]	118.00 [68.00–264.50]	0.79
IV antibiotics within 1 h, NO. (%)	175 (13.10)	129 (14.50)	46 (10.30)	0.04	38 (21.60)	24 (22.00)	14 (20.90)	1.00
IV antibiotics within 3 h, NO. (%)	887 (66.40)	609 (68.40)	278 (62.30)	0.03	103 (58.50)	62 (56.90)	41 (61.20)	0.68
Fluid resuscitation within 3 h, NO. (%)	808 (60.40)	572 (64.20)	236 (52.90)	<0.001	78 (44.30)	46 (42.20)	32 (47.80)	0.41
Time to vasopressor (min)*, median [IQR]	172.00 [81.00–292.00]	168.00 [78.00–280.75]	183.00 [89.00–314.00]	0.07	135.00 [55.00–289.00]	155.50 [59.00–302.50]	107.50 [52.00–256.00]	0.25
Vasopressor within 1 h*, NO. (%)	208 (18.40)	147 (19.00)	61 (17.20)	0.52	46 (28.00)	26 (25.50)	20 (32.30)	0.45
Vasopressor within 3 h*, NO. (%)	590 (52.30)	415 (53.60)	175 (49.30)	0.20	91 (55.50)	53 (52.00)	38 (61.30)	0.32
Time to source control (h)**, median [IQR]	18.62 [7.91–51.18]	18.68 [7.28–55.50]	18.18 [9.16–47.23]	0.56	15.97 [7.22–59.83]	10.93 [6.32–32.72]	30.77 [15.38–482.07]	0.002
Septic shock related interventions	Hospital I				Hospital J			
	Overall (N=688)	Pre-COVID-19 (N=530)	Post-COVID-19 (N=158)	*p*-value	Overall (N=219)	Pre-COVID-19 (N=172)	Post-COVID-19 (N=47)	*p*-value
Time to lactate measurement (min), median [IQR]	23.00 [15.00–49.00]	21.00 [14.00–46.75]	31.00 [19.00–54.50]	<0.001	33.00 [22.00–48.00]	29.00 [21.00–40.25]	42.00 [32.00–82.00]	<0.001
Time to blood culture (min), median [IQR]	46.00 [24.00–172.00]	37.50 [23.00–129.50]	119.00 [38.00–248.75]	<0.001	19.00 [11.00–30.00]	19.00 [11.00–28.00]	17.00 [11.50–53.00]	0.32

Blood culture within 1 h, NO. (%)	383 (56.00)	324 (61.60)	59 (37.30)	<0.001	197 (90.00)	160 (93.00)	37 (78.70)	0.01
Blood culture within 3 h, NO. (%)	520 (76.00)	421 (80.00)	99 (62.70)	<0.001	210 (95.90)	166 (96.50)	44 (93.60)	0.64
Time to IV antibiotics (min), median [IQR]	135.00 [83.00–230.50]	144.00 [91.00–239.00]	102.00 [69.00–196.00]	0.001	158.00 [125.50–227.00]	159.50 [128.00–237.00]	152.00 [106.00–219.00]	0.12
IV antibiotics within 1 h, NO. (%)	95 (13.80)	66 (12.50)	29 (18.40)	0.08	7 (3.20)	2 (1.20)	5 (10.60)	0.01
IV antibiotics within 3 h, NO. (%)	445 (64.80)	332 (62.80)	113 (71.50)	0.05	130 (59.40)	101 (58.70)	29 (61.70)	0.84
Fluid resuscitation within 3 h, NO. (%)	584 (84.90)	450 (84.90)	134 (84.80)	0.20	209 (95.40)	162 (94.20)	47 (100.00)	0.24
Time to vasopressor (min)*, median [IQR]	132.50 [80.00–228.25]	138.00 [82.00–233.00]	121.00 [69.25–210.50]	0.12	126.00 [89.50–180.00]	123.50 [89.00–176.50]	135.00 [99.00–203.50]	0.29
Vasopressor within 1 h*, NO. (%)	117 (18.20)	83 (17.10)	34 (21.50)	0.26	26 (11.90)	22 (12.80)	4 (8.50)	0.58
Vasopressor within 3 h*, NO. (%)	415 (64.40)	311 (64.00)	104 (65.80)	0.75	165 (75.30)	133 (77.30)	32 (68.10)	0.27
Time to source control (h)**, median [IQR]	9.86 [6.36–17.87]	9.28 [5.97–17.71]	14.05 [7.62–19.95]	0.05	22.75 [12.90–42.25]	23.48 [12.82–42.32]	18.97 [17.30–24.63]	0.94
Septic shock related interventions	Hospital K				Hospital L			
	Overall (N=466)	Pre-COVID-19 (N=321)	Post-COVID-19 (N=145)	*p*-value	Overall (N=8)	Pre-COVID-19 (N=8)	Post-COVID-19 (N=0)	*p*-value
Time to lactate measurement (min), median [IQR]	29.50 [19.00–48.00]	28.00 [18.00–45.00]	33.00 [23.00–53.00]	0.01	14.00 [12.75–23.50]	14.00 [12.75–23.50]	-	-
Time to blood culture (min), median [IQR]	76.00 [40.50–148.00]	77.00 [40.00–149.00]	74.50 [41.75–132.50]	0.58	66.00 [33.75–100.25]	66.00 [33.75–100.25]	-	-
Blood culture within 1 h, NO. (%)	182 (39.30)	125 (39.20)	57 (39.60)	1.00	4 (50.00)	4 (50.00)	-	-
Blood culture within 3 h, NO. (%)	383 (82.70)	264 (82.80)	119 (82.60)	1.00	7 (87.50)	7 (87.50)	-	-
Time to IV antibiotics (min), median [IQR]	151.00 [98.50–233.50]	151.00 [99.50–235.00]	151.00 [98.00–222.00]	0.93	88.50 [68.00–124.25]	88.50 [68.00–124.25]	-	-
IV antibiotics within 1 h, NO. (%)	54 (11.70)	43 (13.50)	11 (7.60)	0.09	2 (25.00)	2 (25.00)	-	-
IV antibiotics within 3 h, NO. (%)	277 (59.80)	191 (60.10)	86 (59.30)	0.96	6 (75.00)	6 (75.00)	-	-
Fluid resuscitation within 3 h, NO. (%)	382 (82.00)	259 (80.70)	123 (84.80)	0.26	5 (62.50)	5 (62.50)	-	-
Time to vasopressor (min)*, median [IQR]	141.50 [29.75–235.00]	136.00 [12.50–235.50]	146.00 [60.00–232.00]	0.44	66.00 [42.50–73.00]	66.00 [42.50–73.00]	-	-

Vasopressor within 1 h*, NO. (%)	137 (30.90)	102 (33.20)	35 (25.50)	0.13	3 (37.50)	3 (37.50)	-	-
Vasopressor within 3 h*, NO. (%)	272 (61.30)	188 (61.20)	84 (61.30)	1.00	8 (100.00)	8 (100.00)	-	-
Time to source control (h)**, median [IQR]	7.82 [5.21–16.54]	8.00 [5.18–18.95]	7.57 [5.33–10.58]	0.51	5.75 [5.75–5.75]	5.75 [5.75–5.75]	-	-
Septic shock related interventions	Hospital M				Hospital N			
	Overall (N=8)	Pre-COVID-19 (N=0)	Post-COVID-19 (N=8)	*p*-value	Overall (N=29)	Pre-COVID-19 (N=0)	Post-COVID-19 (N=29)	*p*-value
Time to lactate measurement (min), median [IQR]	41.00 [33.00–56.00]	-	41.00 [33.00–56.00]	-	45.00 [29.00–277.00]	-	45.00 [29.00–277.00]	-
Time to blood culture (min), median [IQR]	177.00 [55.50–278.00]	-	177.00 [55.50–278.00]	-	59.00 [32.00–216.00]	-	59.00 [32.00–216.00]	-
Blood culture within 1 h, NO. (%)	2 (28.60)	-	2 (28.60)	-	9 (52.90)	-	9 (52.90)	-
Blood culture within 3 h, NO. (%)	4 (57.10)	-	4 (57.10)	-	12 (70.60)	-	12 (70.60)	-
Time to IV antibiotics (min), median [IQR]	62.50 [54.50–74.25]	-	62.50 [54.50–74.25]	-	109.00 [69.00–174.00]	-	109.00 [69.00–174.00]	-
IV antibiotics within 1 h, NO. (%)	3 (37.50)	-	3 (37.50)	-	4 (13.80)	-	4 (13.80)	-
IV antibiotics within 3 h, NO. (%)	8 (100.00)	-	8 (100.00)	-	22 (75.90)	-	22 (75.90)	-
Fluid resuscitation within 3 h, NO. (%)	6 (75.00)	-	6 (75.00)	-	18 (62.10)	-	18 (62.10)	-
Time to vasopressor (min)*, median [IQR]	98.00 [53.25–141.25]	-	98.00 [53.25–141.25]	-	193.00 [113.00–313.00]	-	193.00 [113.00–313.00]	-
Vasopressor within 1 h*, NO. (%)	2 (25.00)	-	2 (25.00)	-	4 (16.00)	-	4 (16.00)	-
Vasopressor within 3 h*, NO. (%)	7 (87.50)	-	7 (87.50)	-	10 (40.00)	-	10 (40.00)	-
Time to source control (h)**, median [IQR]	9.78 [9.78–9.78]	-	9.78 [9.78–9.78]	-	12.45 [12.45–12.45]	-	12.45 [12.45–12.45]	-

COVID-19, coronavirus disease 2019; IV, intravenous; * patients required vasopressors; ** patients required source control.
